# Analysis of Codon Usage Patterns in Herbaceous Peony (*Paeonia lactiflora* Pall.) Based on Transcriptome Data

**DOI:** 10.3390/genes6041125

**Published:** 2015-10-22

**Authors:** Yanqing Wu, Daqiu Zhao, Jun Tao

**Affiliations:** Key Laboratory of Crop Genetics and Physiology of Jiangsu Province, College of Horticulture and Plant Protection, Yangzhou University, WenHui East Street 48, Yangzhou 225009, China; E-Mails: yqwu19880928@126.com (Y.W.); daqiuzhao@126.com (D.Z.)

**Keywords:** herbaceous peony (*P*. *lactiflora*), codon usage bias, transcriptome

## Abstract

Codon usage bias, which exists in many genomes, is mainly determined by mutation and selection. To elucidate the genetic features and evolutionary history of herbaceous peony (*Paeonia lactiflora*), a well-known symbol of prosperity in China, we examined synonymous codon usage in 24,216 reconstructed genes from the *P*. *lactiflora* transcriptome. The mean GC content was 44.4%, indicating that the nucleotide content of *P*. *lactiflora* genes is slightly AT rich and GC poor. The *P*. *lactiflora* genome has a wide range of GC3 (GC content at the third synonymous codon position) distribution, with a significant correlation between GC12 and GC3. ENC (effective number of codons) analysis suggested that mutational bias played a major role in shaping codon usage. Parity Rule 2 (PR2) analysis revealed that GC and AU were not used proportionally. We identified 22 “optimal codons”, most ending with an A or U. Our results suggested that nucleotide composition mutation bias and translational selection were the main driving factors of codon usage bias in *P*. *lactiflora*. These results lay the foundation for exploring the evolutionary mechanisms and heterologous expression of functionally-important proteins in *P*. *lactiflora*.

## 1. Introduction

The genetic code is degenerate (64 codons for 20 amino acids and the termination signal), with most amino acids encoded by two to six synonymous codons used at different frequencies, a phenomenon known as codon usage bias (CUB) [1]. Many factors influence codon usage in various organisms, such as natural selection (e.g., gene expression level, tRNA abundance, protein length, gene translation initiation signals and protein structure) and mutational pressure (e.g., GC content, mutation frequency and pattern), as well as random genetic drift [2,3,4]. Codon usage bias plays an important role in predicting the optimum host of exogenous genes and can be used to improve the expression levels of exogenous genes via codon optimization. Codon usage also provides clues about the evolution of different organisms, as well as their environmental adaptation [5].

Genome-wide investigations of codon usage patterns have greatly contributed to our understanding of the basic features of the molecular organization in genomes. To date, most reports about CUB have focused on unicellular and model organisms, such as *Escherichia coli* [6], yeast [7], *Chlamydiae*, *Spirochaete* [8], *Caenorhabditis*, *Drosophila*, *Arabidopsis* [9], *Giardia lamblia* [10], *Entamoeba histolytica* [11] and *Borrelia burgdorferi* [12]. By contrast, few studies have focused on plant species, such as *Paeonia lactiflora* (herbaceous peony). *P*. *lactiflora* is a kind of traditional flower in China belonging to the *Paeoniaceae* family that is a well-known symbol of prosperity. Ornamental plants have broad market appeal, as they stimulate the senses and influence the psychology of consumers [13]. However, since the genome sequence of *P*. *lactiflora* has still not been released, it is difficult to investigate codon usage bias in this species.

Transcriptome sequencing (RNA-Seq) is a highly-efficient, commonly-used research method based on next-generation sequencing technology [14]. We previously obtained transcriptome data for *P*. *lactiflora* using RNA-Seq analysis, leading to the identification of 61,408 unigenes [15]. In the current study, to further analyze the codon usage patterns in the *P*. *lactiflora* genome, we investigated the codon usage profile of *P*. *lactiflora* through analysis of the transcriptome data using multivariate statistical analysis. We analyzed the nucleotide composition of coding sequences from the *P*. *lactiflora* genome, followed by correlation analysis of various factors that influence codon usage bias. The results of this study help elucidate the mechanism underlying the molecular evolution of this species, while providing a theoretical basis for improving the expression levels of exogenous genes by codon optimization.

## 2. Experimental Section

### 2.1. Transcriptome Data

Due to the lack of a complete genome sequence for *P*. *lactiflora*, in this study, 61,408 unigenes previously obtained by transcriptome sequencing were investigated. Based on BLAST (Basic Local Alignment Search Tool) analysis against six public databases, 37,511 unigenes were annotated, including 35,972 in the Non-Redundant Protein database (NR), 30,199 in the Non-Redundant Nucleotide database (NT), 26,674 in the Gene Ontology database (GO), 22,655 in the Swiss-Prot protein database (Swiss-Prot), 20,294 in the Kyoto Encyclopedia of Genes and Genomes database (KEGG) and 13,089 in the Cluster of Orthologous Groups of proteins database (COG). Among all annotated unigenes, a total of 33,281 CDS (coding sequences) were obtained by the BLASTx algorithm [16]. When a unigene was unaligned with any of these databases, ESTScan software was used to determine its sequence direction [17]. To improve the quality of sequences and to minimize sampling errors, all CDS less than 300 bp in length were filtered out. The final sequence collection, containing 24,216 CDS, was used for further analyses.

### 2.2. Indices of Codon Usage

ENC (effective number of codons), which provides useful estimates of absolute codon bias, is a measure that identifies the overall codon use bias for a certain gene. ENC values range from 20 (when only one synonymous codon is chosen by the corresponding amino acid) to 61 (when all synonymous codons are used equally); the lower the ENC value for a single gene, the stronger the overall codon usage bias for this gene [18,19]. The overall GC content, especially the GC3 (GC content at the third position), frequently reflects the strength of directional mutation. RSCU (Relative Synonymous Codon Usage) is an index used to study the overall synonymous codon usage variation among genes [3]. If the RSCU value for a particular codon is equal to 1.0, this codon was chosen equally and randomly. Codons with RSCU values greater than 1.0 have positive codon usage bias, while those with value less than 1.0 have relatively negative codon usage bias. CAI (Codon Adaptation Index) is used to estimate the extent of bias toward codons that are known to be preferred in highly-expressed genes [20]. The higher the CAI value (within a range of 0–1), the stronger the codon usage bias. All CAI values in all 24,216 CDS were assessed using the CodonW program (http://codonw.sourceforge.net) [21].

### 2.3. Neutrality Plot

The neutrality plot is an analytical method used to measure codon usage patterns. In this study, the GC contents at the first, second and third codon positions (GC1, GC2 and GC3, respectively) were analyzed. GC12 represents the average of GC1 and GC2; GC12 and GC3 were used for neutrality plot analysis. In neutrality plots, if the correlation between GC12 and GC3 is statistically significant and the slope of the regression line is close to 1, mutation bias is assumed to be the main force shaping codon usage. Conversely, selection against mutation bias can lead to a narrow distribution of GC content and a lack of correlation between GC12 and GC3 [22].

### 2.4. ENC Plot

The ENC-GC3s plot is widely used to determine whether the codon usage of a gene is affected by mutation and selection [18]. An ENC plot is drawn using the ENC value as the ordinate and the GC3 value as the abscissa. When the corresponding points fall near the expected curve, mutation is the main force shaping codon usage. When the corresponding points fall considerably below the expected curve, selection is the main force shaping codon usage.

### 2.5. Determination of Optimal Codons

Based on the calculated CAI values, 5% of the genes with extremely high and low CAI values were regarded as the high and low datasets, respectively [23]. Codon usage was compared between groups using the chi-square contingency test. Codons whose frequency of usage was significantly higher (*p* < 0.01) in highly-expressed genes than those with low levels of expression were defined as optimal codons.

### 2.6. Correspondence Analysis

The relationships between variables and samples can be explored using multivariate statistical analysis. Correspondence analysis (COA) was performed on RSCU values using CodonW to compare the intra-genomic variation of 59 informative codons partitioned along 59 orthogonal axes (excluding Met, Trp and stop codons) [24].

### 2.7. PR2-Bias Plot Analysis

The nucleotide compositions of the third codon position (A3, U3, C3 and G3) were calculated, and the AU-bias (A3/(A3 + U3)) and GC-bias (G3/(G3 + C3)) were analyzed. Parity Rule 2 (PR2) bias was detected based on the value of AT-bias (A3/(A3 + T3)) as the ordinate and GC-bias (G3/(G3 + C3)) as the abscissa [25].

### 2.8. Statistical Analysis

CodonW1.4.4 software was used to analyze the indices of codon usage. Correlation analyses based on Spearman’s rank correlation (with a level of significance of *p* < 0.05 or *p* < 0.01) were performed with Microsoft Excel and SPSS 18.0 (http://www.spss.com/).

## 3. Results and Discussion

### 3.1. Nucleotide Contents of Genes in P. lactiflora

**Figure 1:**
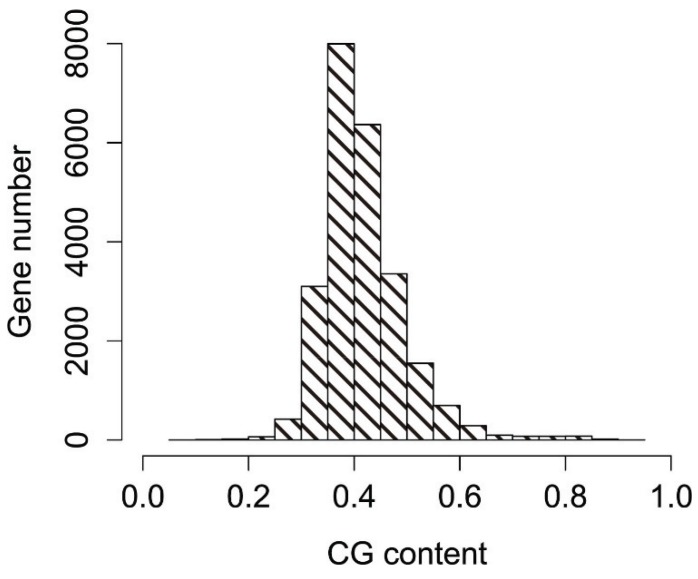
Distribution of the GC contents of reconstructed genes in *P*. *lactiflora*.

Codon usage bias for a single type of codon is greatly influenced by the overall nucleotide content of the genome [26]. Therefore, we firstly analyzed the nucleotide composition of coding sequences (CDS) from the *P*. *lactiflora* genome. In the *P*. *lactiflora* genome, the A nucleotide content ranged from 11.0%–59.9% (mean = 28.44%; SD = 3.64%); the T nucleotide content ranged from 2.6%–58.9% (mean = 27.16%; SD = 3.64%); the C nucleotide content ranged from 3.0%–57.0% (mean = 20.31%; SD = 4.11%); and the G nucleotide content ranged from 3.7%–51.5% (mean = 24.08%; SD = 3.17%). To further examine the nucleotide distribution, we investigated the GC and GC3 contents of reconstructed genes. The GC contents of reconstructed genes ranged from 24.6%–73.6%, with a standard deviation (SD) of 3.91, with most values between 35% and 45% (Figure 1). The mean value of GC contents for the reconstructed genes was 44.4%, suggesting that genes in the *P*. *lactiflora* genome are slightly AT rich and GC poor. The GC content at three codon positions (GC1, GC2 and GC3) was 0.383, 0.304 and 0.418, respectively, demonstrating that the GC content at the third position is different from that at the first and second positions. The GC content at GC1 was extremely close to that at GC2, and the GC content at GC3 was the highest among codon positions. The results from this initial nucleotide composition analysis suggested that A/U-ending codons might be preferred over G/C-ending codons in the *P*. *lactiflora* genome.

**Figure 2:**
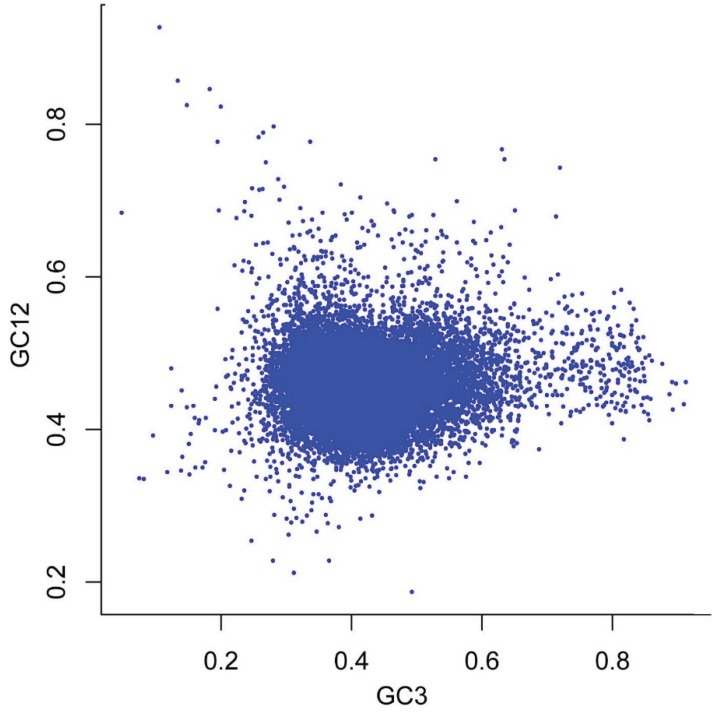
Neutrality plot (GC12 *vs*. GC3).

Neutrality analysis is a useful way to reveal the relationship between GC12 and GC3 and to examine the role of mutation: selection equilibrium in shaping the CUB. To analyze the relationships among the three codon positions, we constructed neutrality plots (GC12 *vs*. GC3) for the *P*. *lactiflora* genome. We found that *P*. *lactiflora* genes had a wide range of GC3 values (5.1–91.6) and that there was a significant correlation between GC12 and GC3 (r = −0.03, *p* < 0.01; Figure 2 and Table 1), suggesting that mutational pressure probably influences codon usage bias in the *P*. *lactiflora* genome.

### 3.2. Relative Synonymous Codon Usage Analysis of the P. lactiflora Genome

To determine the patterns of synonymous codon usage and to what extent A/U-ending codons might be preferred, we performed Relative Synonymous Codon Usage (RSCU) analysis and calculated the RSCU values. Among the 27 most abundantly-used codons in the *P*. *lactiflora* genome, 23 (GCU, AAU, GAU, UGU, GGU, CAU, AUU, CUU, GCA, AGA, CAA, GAA, GGA, UUU, CCA, CCU, AGU, UCA, UCU, ACA, ACU, UAU and GUU) were A/U-ending codons (A-ending: eight; U-ending: 15), and the remaining four (AGG, UUG, AAG and GUG) were G-ending codons; none of the preferred codons were C-ending. These results suggested that compositional limitations played an integral role in the codon usage patterns of *P*. *lactiflora*.

**Table 1:** Correlation analysis of *P*. *lactiflora* gene-related parameters. GC3, GC content at the third synonymous codon position. CAI, Codon Adaptation Index.

Parameters	GC12	GC3	GCall	*ENC*	*CAI*
GC3	−0.03^**^				
GCall	0.737^**^	0.590^**^			
*ENC*	0.100^**^	−0.053^**^	0.034^**^		
*CAI*	−0.107^**^	0.330^**^	0.124^**^	−0.405^**^	
*Axis1*	0.159^**^	0.758^**^	0.610^**^	0.022^**^	0.293^**^

* Significant difference at *p* < 0.05; ** significant difference at *p* < 0.01.

### 3.3. Determination of Codon Usage Bias Based on ENC

**Figure 3:**
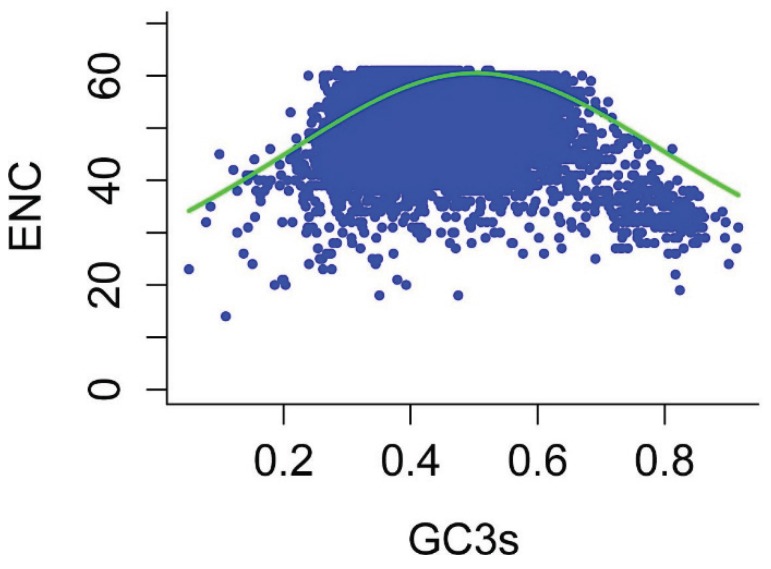
Relationship between GC3 and the effective number of codons (ENC) (ENC plot). ENC is plotted against GC content at the third codon position. The expected ENC from GC3 is shown as a standard curve.

The effective number of codons (ENC) is widely used to measure the codon bias levels of individual genes. The ENC values of the reconstructed genes ranged from 14–61, indicating that there are significant differences in codon bias among these genes. To elucidate the relationship between nucleotide composition and codon bias in *P*. *lactiflora* sequences, we plotted ENC and GC3s, thereby exploring the main features of codon usage among genes [18]. As shown in Figure 3, most points were aggregated close to the expected ENC curve, although there were some points with low ENC below the expected curve, indicating that in addition to mutational pressure, the codon usage patterns are also influenced by other factors (e.g., translational selection) to some extent.

**Figure 4:**
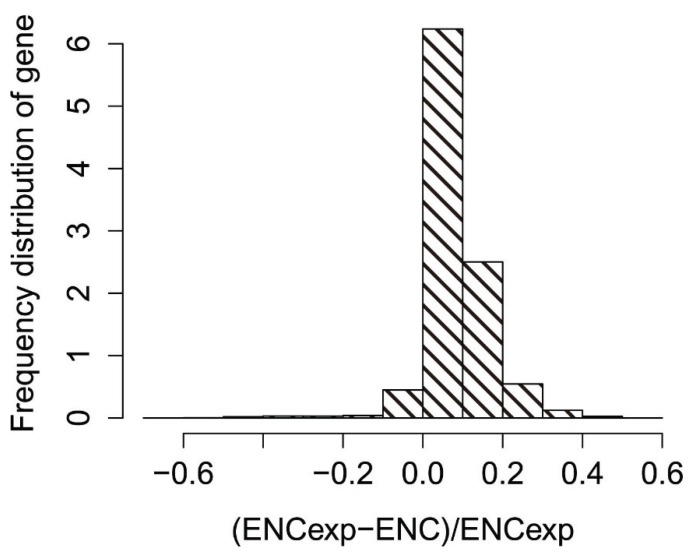
Frequency distribution of the effective number of codons (ENC) ratio.

To obtain a more accurate estimate of the differences between observed and expected ENC values, we calculated (ENCexp-ENCobs)/ENCexp. As shown in Figure 4, the peak (ENCexp-ENCobs)/ENCexp value was 0–0.1, and most genes had (ENCexp-ENCobs)/ENCexp values of −0.1–0.3, indicating that most genes have ENCs slightly different from the expected ENC values based on their GC3s. Therefore, most genes have observed ENCs close to the expected ENC based on GC3s, although a significant number have much lower observed ENCs.

### 3.4. Correspondence Analysis

**Figure 5:**
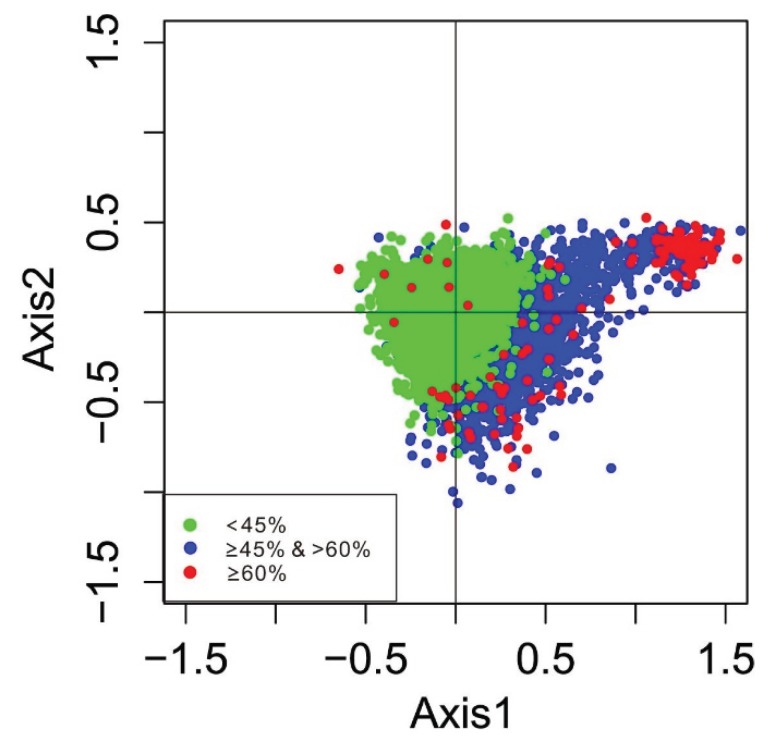
Correspondence analysis of codon usage patterns: the distribution of genes is shown along the first and second axes. Note: red, green and blue dots indicate genes with G + C content greater than or equal to 60%, greater than or equal to 45%, but less than 60%, and less than 45%, respectively.

**Figure 6:**
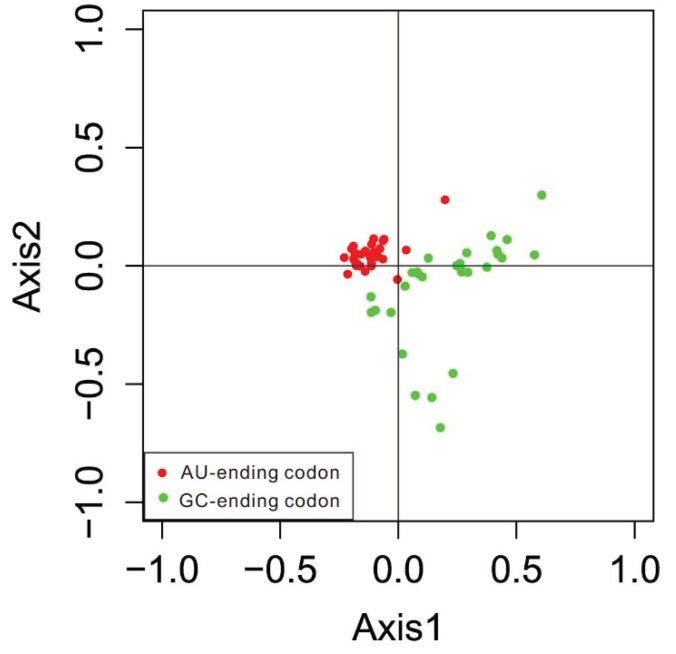
Correspondence analysis of codon usage patterns: the distribution of codons on the same two axes is shown.

Since codon usage is multivariate by nature, it is important to analyze codon usage data using multivariate statistical techniques, such as correspondence analysis (COA) [24]. We determined that the four main contributors were Axis 1, Axis 2, Axis 3 and Axis 4. The first two main dimensional coordinates, Axis 1 and Axis 2, explain 20.55% and 13.99% of the total variance, respectively, while Axis 3 and Axis 4 explain 12.24% and 11.45% of variance, respectively, indicating that the first axis is the major contributor to codon bias. To illustrate the effects of the GC contents of genes on codon usage bias, the GC contents of genes are color-coded on the plot (Figure 5). Genes with a GC of 60% were plotted as red triangles, while genes with a GC less-than 45% were plotted as green dots. Blue dots indicated genes with a GC content between 45% and 60%. The results showed that high and low GC contents of genes could be separated along the primary axis. Figure 6 shows the separation of different base-ending codons along the two axes. The separation of codons on the first axis appears to be largely due to frequency differences between G/C- and A/T-ending codons.

To identify the main contributors to codon bias, we calculated the correlations among the most important indices (Table 1). Significant correlations were found among indices. Axis 1 showed significant correlations between GC3, Codon Adaptation Index (CAI) and ENC (r = 0.758, *p* < 0.01; r = 0.293, *p* < 0.01; r = 0.022, *p* < 0.01), suggesting that the base composition for mutation bias (r = 0.758) and translational selection (r = 0.293) might have an impact on codon bias.

### 3.5. PR2-Bias Plot Analysis

To investigate whether the biased codon choices are restricted to highly-biased protein-coding genes, we analyzed the association between purines (A and G) and pyrimidines (C and T) in four-codon amino acid families by a Parity Rule 2 (PR2) bias plot [27], finding that G and T are used more frequently than A and C in *P*. *lactiflora*. Differences between C/G and A/T contents were commonly observed in most protein-coding genes, suggesting that mutation bias mainly contributes to codon bias.

### 3.6. Effects of Gene Expression Level and Encoded Protein Length on Synonymous Codon Usage Bias

**Figure 7:**
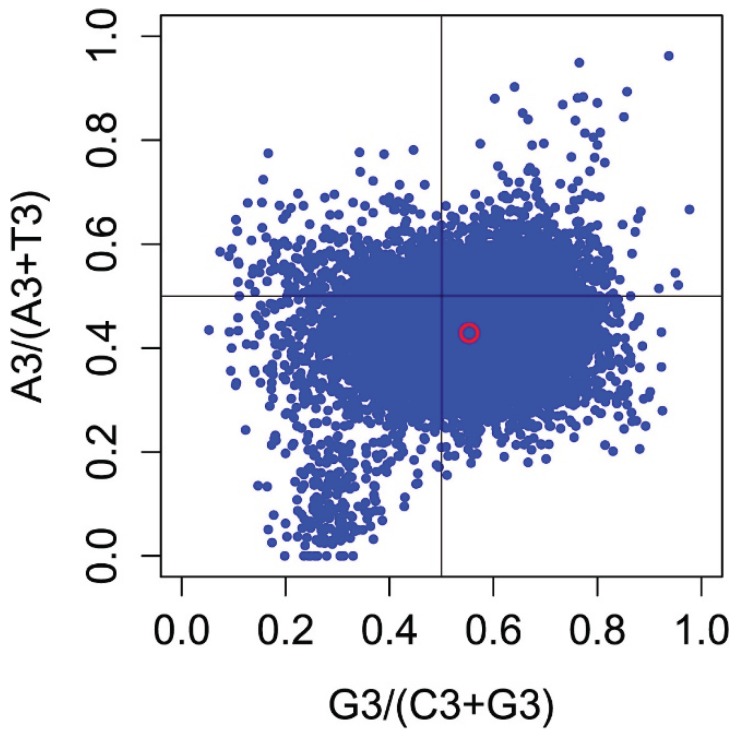
Parity Rule 2 (PR2)-bias plot A3/(A3 + T3) against G3/(G3 + C3). Note: the red open circle indicates the average position for each plot, calculated as follows: x = 0.5534077 ± 0.09976, y = 0.4288815 ± 0.07275.

CAI is commonly used as a predictor of gene expression level [28,29]. To explore the correlation between codon usage bias and gene expression level in *P*. *lactiflora*, we calculated the correlation coefficient between CAI and nucleotide composition/ENC. As shown in Figure 7 and Table 1, we identified one significantly negative correlation between the gene expression level assessed by CAI and ENC values (r = −0.405, *p* < 0.01), as well as two significantly positive correlations between the CAI value and GC3s and GC content (r = 0.330 and 0.124, respectively, *p* < 0.01).

**Figure 8:**
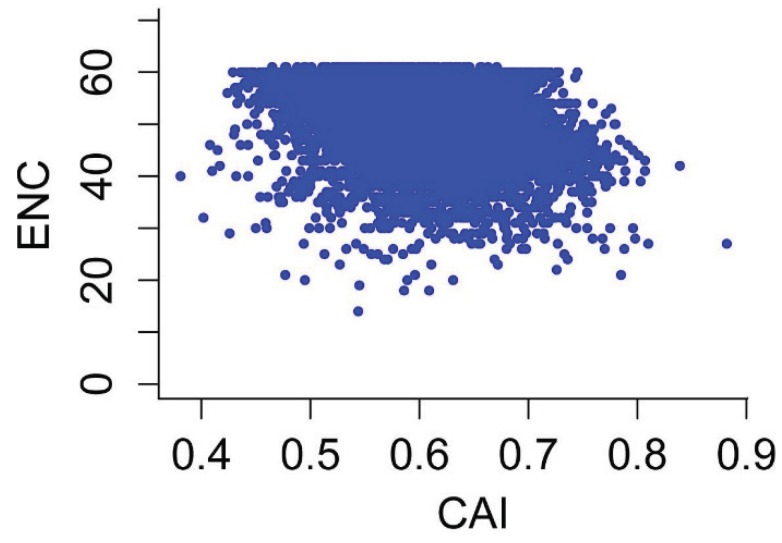
Plot of ENC *vs*. gene expression level for *P*. *lactiflora*.

**Figure 9:**
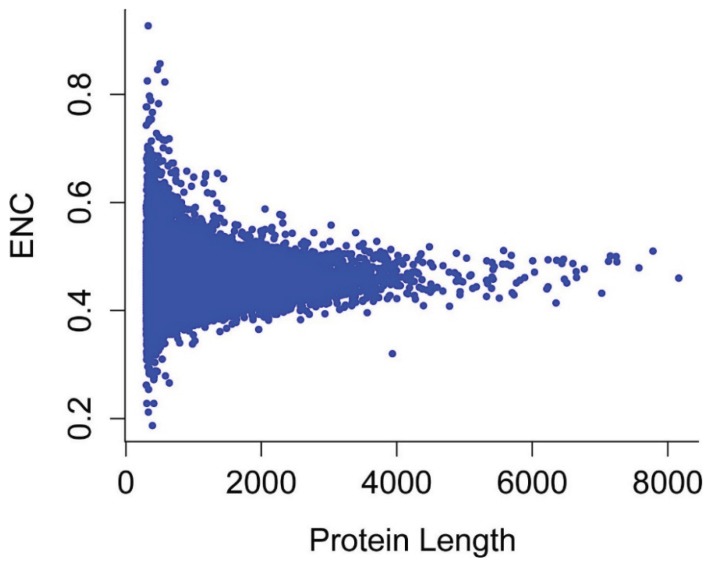
Plot of ENC *vs*. encoded protein length for *P*. *lactiflora*.

Correlation analyses between protein length and Axis 1 coordinates, ENC and CAI values showed that these values are significantly correlated, with correlation coefficients of r = −0.077, 0.906 and −0.438, respectively (*p* < 0.01; Figure 8 and Figure 9), suggesting that in general, more biased genes with shorter lengths have higher expression levels.

### 3.7. Optimal Codons

The average RSCU values of sample groups of genes with high/low expression levels are listed in Table 2. We determined that 22 codons were optimal codons; these codons were significantly more frequent among the highly-expressed genes (*p* < 0.01) according to the chi-square test. Most optimal codons (except GCC, UGC, AUC, UUC, CCC, UCC, UCG, UAC and GUG) end with an A or T, suggesting that codon usage in *P*. *lactiflora* is biased to A- or U-ending synonymous codons.

In this study, we analyzed the codon usage patterns in *P*. *lactiflora* based on a transcriptome dataset from *P*. *lactiflora* derived by deep sequencing. We excluded CDS with gaps and those less than 300 bp in size, yielding 24,216 reconstructed genes from a total of 61,408 non-redundant unigene sequences. We used FrameDP and BLASTx to generate training sequences and calculated training matrices, which greatly improved the quality of the sequences. To date, the whole *P*. *lactiflora* genome has not been sequenced. Therefore, our reconstruction of 24,216 genes comprises a representative sample of *P*. *lactiflora* for codon usage analysis. Codon usage bias is an important, complex phenomenon that continues to evolve. Previous studies have focused on the relationship between gene expression and CUB in prokaryotic organisms and lower eukaryotes [30]. Subsequent studies have focused on CUB in mammals and plants, as well as the relationship between CUB, gene expression patterns and molecular evolution [5]. From a theoretical viewpoint, synonymous codon usage patterns at least partially account for mutation: selection equilibrium between various synonymous codons in specific organisms [31]. While mutation and selection are the main forces shaping codon usage, many other factors also influence CUB, including gene expression [32], nucleotide compositional constraint [33], GC content [34], RNA structure [35], protein length and structure [36], intron length [37], and so on. In the current study, we found that mutational pressure is responsible for nucleotide composition in shaping the strength of codon usage; meanwhile, translational selection mediated by gene expression level was also the main factor shaping the codon usage pattern.

**Table 2:** Comparison of codon usage frequencies between *P*. *lactiflora* sequences with high and low levels of expression.

AA	Codon	High RSCU(N)	Low RSCU(N)	AA	Codon	High RSCU(N)	Low RSCU(N)
Ala	GCA	1.245(3868)	1.296(10,358)	Leu	CUA	0.637(1889)	0.510(3679)
	GCC^*^	0.888(2761)	0.610(4878)		CUC	1.036(3071)	0.662(4778)
	GCG	0.471(1464)	0.305(2439)		CUG	0.757(2244)	0.671(4848)
	GCU^*^	1.396(4338)	1.789(14,297)		CUU^*^	1.343(3981)	1.621(11,707)
Arg	AGA^*^	1.582(2545)	1.939(8781)		UUA	0.838(2486)	0.893(6449)
	AGG	1.478(2378)	1.552(7025)		UUG	1.389(4119)	1.643(11,862)
	CGA	0.831(1338)	0.641(2901)	Lys	AAA	0.911(6111)	0.925(13,915)
	CGC	0.582(936)	0.481(2177)		AAG	1.089(7299)	1.075(16,161)
	CGG	0.648(1042)	0.492(2228)	Met	AUG	1.000(4764)	1.000(10,846)
	CGU	0.880(1416)	0.895(4054)	Pro	CCA^*^	1.376(3278)	1.458(8919)
Asn	AAC	0.882(3785)	0.627(6592)		CCC^*^	0.779(1855)	0.590(3608)
	AAU	1.118(4801)	1.373(14,443)		CCG	0.606(1444)	0.383(2340)
Asp	GAC	0.742(3791)	0.529(7071)		CCU	1.239(2953)	1.569(9600)
	GAU	1.258(6427)	1.471(19,638)	Ser	AGC^*^	0.807(2057)	0.573(4176)
Cys	UGC^*^	0.949(1812)	0.748(2950)		AGU	1.031(2628)	1.074(7834)
	UGU^*^	1.051(2005)	1.252(4937)		UCA^*^	1.218(3105)	1.427(10,405)
Gln	CAA	1.086(3875)	1.099(10,044)		UCC^*^	0.961(2450)	0.670(4885)
	CAG	0.914(3259)	0.901(8230)		UCG^*^	0.620(1580)	0.431(3144)
Glu	GAA	1.038(6811)	1.112(18,684)		UCU	1.365(3481)	1.826(13,318)
	GAG	0.962(6315)	0.888(14,916)	Thr	ACA^*^	1.198(2842)	1.316(7458)
Gly	GGA^*^	1.195(3801)	1.270(10,495)		ACC	1.047(2485)	0.745(4221)
	GGC	0.733(2333)	0.546(4515)		ACG	0.491(1166)	0.365(2069)
	GGG	0.849(2699)	0.779(6443)		ACU	1.263(2997)	1.574(8922)
	GGU	1.223(3890)	1.405(11,615)	Trp	UGG	1.000(2675)	1.000(5315)
His	CAC	0.871(2065)	0.609(3419)	Tyr	UAC^*^	0.917(2611)	0.702(4462)
	CAU	1.129(2679)	1.391(7818)		UAU^*^	1.083(3082)	1.298(8257)
Ile	AUA^*^	0.765(2676)	0.730(5857)	Val	GUA^*^	0.711(2143)	0.642(5096)
	AUC^*^	0.939(3286)	0.632(5066)		GUC	0.801(2412)	0.569(4523)
	AUU	1.296(4532)	1.638(13,133)		GUG^*^	1.106(3331)	0.930(7389)
Phe	UUC^*^	0.939(4017)	0.665(6011)		GUU	1.382(4163)	1.859(14,764)
	UUU^*^	1.061(4535)	1.335(12,077)				

Note: AA: amino acid; N: number of codons; RSCU: Relative Synonymous Codon Usage. Codon usage was compared using a chi-square test to identify optimal codons. * Codons that occur significantly more often (*p* < 0.01).

Nucleotide composition is one of the most important factors shaping codon usage among genes and genomes. The synonymous codon usage bias responds to the AT- or GC-rich content of the genome, while the third position of a codon is considered to be the most likely position reflecting genome base composition. Recently, GC-rich organisms, such as *Triticum aestivum*, *Hordeum vulgare* and *Oryza sativa* [38], have been shown to prefer to use G or C at the third position. Moreover, AT-rich organisms show a preference for A or T at the third position, such as *Plasmodium falciparum*, *Schistosoma* and *Mycoplasma capricolum* [39,40,41]. The mean GC (44.4%) and GC3 (41.8%) contents of the *P*. *lactiflora* genome, as determined in the current study, suggest that this genome is slightly A + T rich, and the overall codon usage is biased toward A- and T-ending codons.

Neutrality analysis revealed a significant correlation between GC12 and GC3 in the *P*. *lactiflora* genome, which indicates that the nucleotide composition of the third codon position (GC3) is slightly different from that of the first and second codon positions. Moreover, the nucleotide at the third codon position has been strongly influenced by mutational pressure. Finally, the first axis (the major contributor to codon bias) was significantly correlated with GC3 (r = 0.758, *p* < 0.01), further suggesting that mutational pressure (r = 0.758) probably plays an important role in codon usage bias in the *P*. *lactiflora* genome.

The ENC plot revealed the expected positions of genes whose codon usage was only determined based on variations in GC3 content. Most of the corresponding points were located near the solid curve of this distribution, whereas only a few points considerably departed from this curve, which indicates that the codon usage of most *P*. *lactiflora* genes is influenced by mutation, and only a few genes are influenced by selection. CAI is widely used to examine the expression levels of genes and is considered to be a well-accepted measure of natural selection [28,29]. Correlation analyses between the first axis and CAI values showed a significant correlation (r = 0.293, *p* < 0.01), further suggesting that translational selection also influences the codon usage bias of the *P*. *lactiflora* genome to some extent. We investigated whether the codon usage of *P*. *lactiflora* genes was also subject to other factors, finding that the ENC value of each gene is significantly correlated with its GC3 content, GC content, protein length and CAI value. These findings indicate that nucleotide composition, protein length and gene expression level shape synonymous codon usage bias in *P*. *lactiflora*.

In this study, 22 codons were identified as optimal codons. Most of the optimal codons in the *P*. *lactiflora* genome end with an A or T, which is similar to the pattern observed in other eukaryotic genomes, such as *Plasmodium falciparum* [39], *Schistosoma* [40] and *Mycoplasma capricolum* [41]. These results provide a useful reference for genetic engineering and evolutionary studies.

## 4. Conclusions

We investigated the pattern of codon usage bias in the *P*. *lactiflora* genome, as well as its causative factors. The mean GC content (44.4%) and GC3 (41.8%) in the *P*. *lactiflora* genome indicate that the nucleotide content of genes is slightly AT rich and GC poor, and overall codon usage is biased toward A- and T-ending codons. We found that the codon usage pattern in *P*. *lactiflora* genome was significantly (*p* < 0.01) influenced by major factors, such as nucleotide composition mutation (r = 0.758) and translational selection (r = 0.293). In addition, 22 optimal codons were identified, all of which ended with either an A or T residue. These findings will be useful for cloning and expressing exogenous genes in this plant. In conclusion, a series of comprehensive analyses of synonymous codon usage patterns has provided a basic understanding of the mechanisms underlying codon usage in *P*. *lactiflora*. This information could be helpful in future investigations of evolutionary mechanisms in *P*. *lactiflora*, as well as gene cloning and heterologous expression of functionally-important proteins.
